# The cellular dynamics of neural tube formation

**DOI:** 10.1042/BST20220871

**Published:** 2023-02-16

**Authors:** Marise van der Spuy, Jian Xiong Wang, Dagmara Kociszewska, Melanie D. White

**Affiliations:** Institute for Molecular Bioscience, The University of Queensland, Brisbane, QLD 4072, Australia

**Keywords:** developmental biology, embryogenesis, neurodevelopment

## Abstract

The vertebrate brain and spinal cord arise from a common precursor, the neural tube, which forms very early during embryonic development. To shape the forming neural tube, changes in cellular architecture must be tightly co-ordinated in space and time. Live imaging of different animal models has provided valuable insights into the cellular dynamics driving neural tube formation. The most well-characterised morphogenetic processes underlying this transformation are convergent extension and apical constriction, which elongate and bend the neural plate. Recent work has focused on understanding how these two processes are spatiotemporally integrated from the tissue- to the subcellular scale. Various mechanisms of neural tube closure have also been visualised, yielding a growing understanding of how cellular movements, junctional remodelling and interactions with the extracellular matrix promote fusion and zippering of the neural tube. Additionally, live imaging has also now revealed a mechanical role for apoptosis in neural plate bending, and how cell intercalation forms the lumen of the secondary neural tube. Here, we highlight the latest research on the cellular dynamics underlying neural tube formation and provide some perspectives for the future.

## Introduction

The formation of the neural tube is a critical process in the embryonic development of vertebrates. It is rapid and complex, involving genetic, morphogenetic, epigenetic, mechanical, and environmental cues. During neurulation, multiple cellular processes are tightly co-ordinated in time and space to convert the flat neural plate into the neural tube, which will give rise to the central and peripheral nervous systems [1,2]. The process of vertebrate neurulation is variable among species, mainly in regard to the number and timing of closure points as well as the contributions of primary and secondary neurulation [3]. Primary neurulation shapes the anterior part of the neural tube and is highly conserved amongst a variety of vertebrate organisms including zebrafish (*Danio rerio*), frogs (*Xenopus laevis*), avian species such as chick (*Gallus domesticus*) and quail (*Coturnix japonica*) and mammalian species including mouse and human [4–10]. Although the details vary between species, the general process of primary neurulation involves remodelling of the neural plate by convergent extension, bending of the tissue to create the neural folds and fusion of the apposed neural folds to form a tube [3,4]. The posterior end of the neural tube is generated through a process of secondary neurulation. Formation of the secondary neural tube results from aggregation and mesenchyme-to-epithelial transition of a loosely packed group of cells in the posterior neural plate. This forms a condensed rod of tightly adherent, polarised epithelial cells which cavitates to form a neuroepithelium surrounding a lumen [11]. Despite the substantial morphological variation in neurulation morphology between vertebrate species, significant similarity and conservation is maintained in the underlying molecular and cellular mechanisms [3].

Incorrect neural tube formation causes severe congenital malformations called neural tube defects (NTDs) which are amongst the most common birth defects. Approximately 300 000 babies are born every year with NTDs leading to ∼88 000 deaths and 8.6 million years of life lost due to disability and premature death annually [12].

## Cellular dynamics of development

Neural tube morphogenesis requires the precise spatiotemporal coordination of changes in cellular shape and position. These dynamic cellular changes are primarily generated by the processes of convergent extension, apical constriction and cell intercalation. Failures in any of these processes disrupts the morphology of the neural tube and may result in NTDs. To understand how these dynamic processes interact across scales to form the neural tube, it is necessary to image and manipulate them in real time in the living embryo. Here, we will discuss recent work using high resolution live imaging approaches in different model organisms to understand the cellular dynamics of neural tube formation.

### Convergent extension

**Figure 1.**
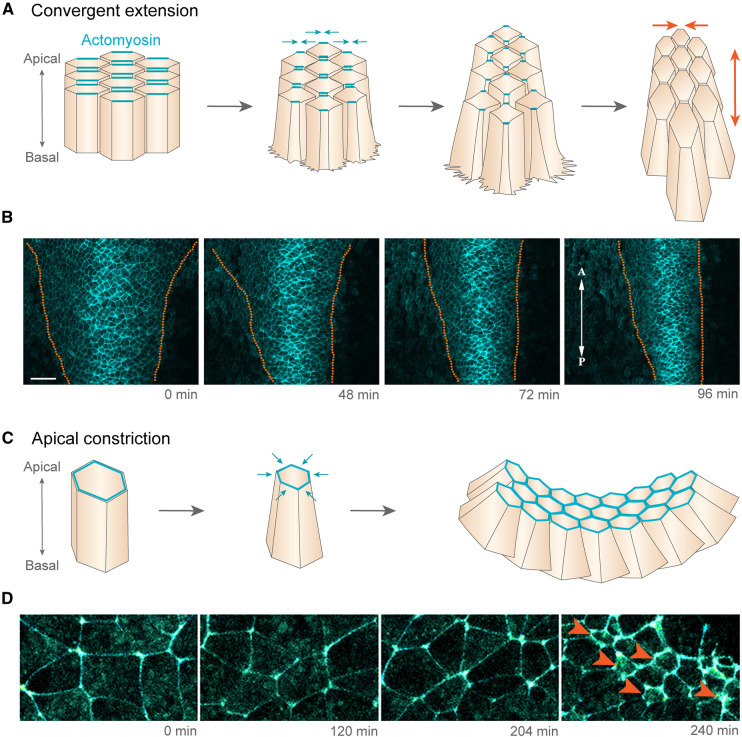
Convergent extension and apical constriction in neural tube morphogenesis. (**A**) Schematic showing how convergent extension drives the narrowing and lengthening of the neural plate. (**B**) Stills from time-lapse recordings of *Xenopus* neurula stage embryos expressing membrane-GFP. Dotted lines delineate the boundaries of the neural plate and show narrowing of the neural plate and neural fold movement towards the midline. (**C**) Schematic showing how apical actomyosin contraction reduces the apical cell surface area to achieve apical constriction and bend an epithelial tissue. (**D**) Stills from a time-lapse recording from the posterior region of a neurula-stage *Xenopus* embryo. Arrowheads indicate the contraction of apical actomyosin and reduced apical cell surface area during AC. **B** and **D** adapted with permission from [23].

Convergent extension (CE) is a morphogenetic process in which tissue narrows or converges along one axis and elongates or extends in one or both of the orthogonal axes (Figure 1). CE is a fundamental mechanism that shapes many different tissues in both vertebrates and invertebrates and is critical for neural tube formation [13]. One of the most ubiquitous mechanisms underlying CE is polarised cell intercalation directed by the noncanonical Wnt/Planar Cell Polarity (PCP) pathway. PCP signalling polarises cells within the plane of the tissue and perpendicular to the apicobasal axis [14]. It relies on the asymmetric segregation of modules of core PCP proteins to opposite axial domains of the cell cortex in a pattern that propagates throughout the tissue. A major target of PCP signalling is regulation of the actin cytoskeleton. Unlike the canonical Wnt/β-catenin-induced gene regulation pathway, the noncanonical Wnt/PCP pathway exerts functions on cellular polarisation and downstream directional actomyosin remodelling [15]. Although the Wnt/PCP pathway was originally identified in wing and eye development in *Drosophila* embryos [16,17], the orthologous genes and proteins are conserved across a variety of vertebrates such as *Xenopus*, zebrafish, avian animals, mouse, and human [18–22], confirming its fundamental role in development.

A high proportion of the genetic mutations that have been linked to NTDs are found in members of the PCP signalling pathway [24], and recent advances in live imaging approaches are providing insight into the critical dynamic functions of PCP proteins in neural tube morphogenesis. Live imaging of the *Xenopus* neural plate epithelium revealed that differential turnover of the PCP proteins Prickle2 (Pk2) and Vangl2 leads to their active enrichment specifically at shrinking cell junctions [25]. Futhermore, this junctional accumulation of Pk2 is pulsatile and spatiotemporally correlated with pulsatile enrichment of actomyosin. Disrupting PCP signalling not only perturbs asymmetric Pk2 protein localisation, but also inhibits the planar polarisation of actomyosin contraction and CE in the closing neural tube. Computer simulations suggest that asynchronous actomyosin contractions that alternate at an optimal frequency between neighbouring cells underly efficient CE [26]. Interestingly, Pk2 junctional localisation is also pulsatile in the *Xenopus* mesoderm, and correlates with oscillations of actomyosin that are required for CE. Pk2 tunes the frequency of these actomyosin oscillations suggesting that temporal asymmetry of PCP proteins at cell junctions may be as important as their planar asymmetry [26].

As the core PCP protein, Frizzled, functions as a Wnt receptor [27], Wnt signalling has long been proposed to control planar cell polarity, yet multiple overlapping inputs now appear to influence PCP establishment and orientation [28]. Within the *Xenopus* neuroectoderm, ectopic Wnt expression can reorient the polarity of the core PCP protein Vangl2, suggesting that a gradient of Wnt signals from the posterior of the embryo may establish planar cell polarity along the anterior–posterior axis of the neural tube [29]. However, mechanical cues such as actomyosin contraction and actin remodelling have also been shown to instruct planar cell polarity [30,31]. Recent work applying uniaxial stretch to *Xenopus* explants reveals a cooperative relationship between Wnt signalling and mechanical inputs in the neuroectoderm [32]. Both Wnt signalling and unidirectional tension can control the orientation of PCP, however the degree of polarisation is greatest when the direction of the mechanical input is aligned with a diffusion gradient of Wnt signalling. This suggests a model where tissue stretch along the anterior–posterior axis arising from morphogenic movements coincides with a posterior Wnt diffusion gradient to reinforce the robustness of PCP in the developing neural tube. These findings are beginning to reveal how neural tube morphogenesis is directed by a complex interplay between cellular properties and mechanical and biochemical cues across scales.

Two distinct modes of CE, namely cell crawling and cell junction contraction, have previously been identified as contributing to CE cell movements (reviewed by [33]). In the mouse neural plate, these two processes can occur concurrently within cells during CE [34]. Recent work combining live imaging of *Xenopus* embryo mesoderm with computational modelling showed that although cell crawling and junction contraction can occur both independently and collaboratively throughout development, CE is more efficient when these processes are integrated within the same cell [35]. Actin assembly associated with cell crawling and junction contraction is augmented when these processes occur concurrently, significantly increasing the efficiency of CE. This synergistic effect resembles the feedforward loop that promotes actomyosin cable formation in *Drosophila* [36], suggesting that mechanoreciprocity between the two pools of actomyosin may integrate cell crawling and junction contraction. Whether and how these two modes of CE also contribute to neural tube formation in other organisms remains to be determined. To date, most live imaging of CE during neural tube formation has been performed using *Xenopus* but a recent study in mice suggested the process appears to be mostly conserved in the mammalian model [34]. However, recent advances in transgenic avian models offer an exciting system for real-time investigation of cellular, morphogenetic, genetic and signalling dynamics underlying neural tube morphogenesis in a higher vertebrate [37,38].

### Apical constriction

Apical constriction (AC) is a common mechanism of tissue remodelling that involves reduction in the apical surface area of a cell [39]. The resulting changes in cell geometry can shape tissues by bending epithelial sheets or causing cell ingression or extrusion (Figure 1). To this end, AC plays a part in neurulation by contributing to the formation of the ‘bending points’ which facilitate the bending of the neural plate and also assists in force generation which promotes closure of the neural tube [3]. However, these bending points or hinge points may not be exclusively generated through AC. Recent computational modelling suggests that the formation of the dorso-lateral hingepoints in the spinal neural tube could be a passive response to the zippering process during neural tube closure [40].

The pseudostratified epithelial cells of the neural tube undergo interkinetic nuclear migration as a function of the cell cycle. Subsequently, cells in the S phase tend to have smaller apical surfaces due to basally located nuclei. Both AC and interkinetic nuclear migration have been described as important for neural tube closure [41,42]. Recently, live imaging of the mouse neural plate demonstrated that there is coordination between these two processes, specifically in the posterior neural pore (PNP) [43]. While the natural progression of internuclear migration has widening effects on the PNP, Rho kinase (ROCK) acts to compensate by maintaining tension in the neuroepithelium and facilitating AC for the progression of neural tube closure. Inhibiting ROCK, with specific inhibitor Y27632, causes an increase in apical area and a reduction in PNP tension, suggesting an indispensable role for ROCK-mediated AC in mouse PNP closure.

AC is driven by actomyosin contraction and studies in frog and chick embryos have described pulsed medial actomyosin-based contractions occurring during neural tube closure (reviewed in [39]). In *Xenopus*, distinct patterns of AC behaviour have been observed in the anterior and posterior regions of the neural plate during neural tube closure [44]. In the anterior neural ectoderm, a greater proportion of cells display AC and cells undergo a gradual reduction in apical surface area. However, neural ectoderm cells in the posterior neural plate display later and more rapid apical area reductions. Intriguingly, both N-cadherin and actin accumulate at cell junctions and the medial cell surface in the anterior neural ectoderm, but N-cadherin did not accumulate in the posterior neural ectoderm cells. This differential behaviour could reflect a region-specific function of Shroom3 in coupling actin dynamics to N-cadherin in the anterior neural ectoderm, since the loss of Shroom3 results in a reduced accumulation of medial and junctional actin and decreased capacity for AC specifically in this region. In the posterior neural ectoderm, Shroom3 seems to control polarisation of junction contractions underlying CE without affecting AC, suggesting an interaction with the PCP pathway. These different functions of Shroom3 may also correspond to a previous observation that inactivation of Shroom3 leads to highly penetrant cranial NTDs but weakly penetrant spinal NTDs [45]. Together, these findings demonstrate that distinct mechanisms couple cell shape changes to actomyosin and cell adhesion along the anterior–posterior axis of the embryo.

### Integration of convergent extension and apical constriction

**Figure 2.**
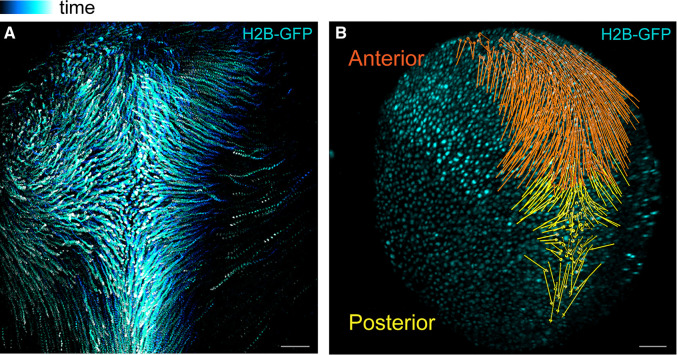
The neural plate displays differential anterior–posterior behaviour. (**A**) Representative temporal colour-coded maximum intensity projection of a time-lapse recording from a *Xenopus* embryo expressing H2B-GFP. (**B**) Displacement map of single-cell tracks overlaid over H2B-GFP signal at t0. The posterior neural plate cells move towards the midline. Anterior neural plate cells move anteriorly and ventrally. Adapted with permission from [23].

The processes of CE and AC are the two most prominent morphogenetic movements active in the neural plate and both processes are indispensable to neural tube closure [42,46,47]. However, they do not operate in isolation and recent work applying live imaging approaches is beginning to reveal how differing spatiotemporal dynamics of AC and CE are integrated to form the neural tube (Figure 2) [23]. In *Xenopus*, the distinct cell behaviours, visualised through microinjections of histone-GFP mRNA, in the anterior and posterior regions of the neural plate may be due to differential PCP activity. Although PCP-mediated cell intercalation and CE movements are restricted to the posterior neural plate during the first phase of neural tube closure, the force this generates is required for elongation of the anterior neural plate. Physical coupling between the anterior and posterior neural plate facilitates the ability of posterior CE to generate forces which affect the anterior neural plate. Subsequently, AC is initiated throughout the entire neural plate with no apparent temporal overlap with CE.

In mice, the scaffold protein Scribble maintains cell junction composition and mediates junctional remodelling to co-ordinate both apical-basal and planar cell polarity [48]. By regulating tight and adherens junctions, Scribble directs actomyosin dynamics to integrate CE and AC. Scribble mutations are associated with defects in both CE and AC, where mediolateral convergence and columnar-to-wedge shape conversion fail in these two processes, respectively. Using live imaging to track the frequency and polarity of cell intercalation in Scribble mutant mouse embryos showed that Scribble regulates the proportion of cells that undergo anterior–posterior versus mediolateral intercalation. This suggests a model where Scribble contributes to determination of the polarity of cell intercalations while also promoting the formation of cellular rosettes, possibly through the mediation of junctional remodelling. Although direct evidence is still lacking, Scribble may regulate cell shape changes and intercalation through Rho-mediated actomyosin pulsations. Intriguingly, mice with mutations in Scribble (*Scrib^rumz^*) or the PCP proteins Vangl2 (*Vangl^Lp^)* and Ptk7 (*Ptk7^XST8^*) all have defects in AC and the frequency or polarity of CE, however only the *Scrib^rumz^ and Ptk7^XST8^* show significantly decreased CE of the entire body axis. This suggests that mediolateral cell intercalation co-operates with AC and further unidentified mechanisms to generate the force required for axis elongation and neural plate shaping.

## Additional morphogenetic mechanisms

### Neural tube closure

Closure of the neural tube occurs concurrently in amphibians but at multiple sites in mammals [3]. Historically, two evolutionarily conserved neural tube closure mechanisms have been proposed: the ‘purse-string model’ and the ‘cell-crawling model’ [49]. It is now accepted that a combination of the two models is important for neural tube closure, with significant differences in cellular dynamics between caudal and rostral regions of the embryo. In mice, live imaging of neural tube closure showed that a combination of both purse-string contractility and directional cell movement in the overlying surface ectoderm (‘cell crawling’) are required to achieve hindbrain neuropore (HNP) closure [50]. Actomyosin purse strings form around the HNP and colocalise with E-cadherin in surface ectoderm, suggesting that surface ectoderm cells establish the first points of contact. Subsequent closure of the HNP proceeds asymmetrically with faster closure in the rostral-to-caudal direction. Simulations indicate that this is due to an increased radius of curvature at the caudal end of the gap which constrains closure. This powerful combination of live imaging and modelling has provided new insights into how the underlying tissue geometry influences actomyosin purse-string contraction and cell crawling to drive the cellular dynamics of neural tube closure.

An additional mechanism of neural closure recently identified in the mouse PNP is integrin-mediated anchorage [51]. Although integrin β1 is known to be important for neural tube closure, its exact role remained unclear [52,53]. Recently, live imaging of mouse PNP closure suggested that the initial adhesion between surface ectoderm cells from apposing epithelial layers requires integrin β1 at zippering sites. As the neural folds elevate and become apposed, a basement membrane rich in fibronectin is deposited between the dorsal tips of the neural folds and the overlaying surface ectoderm. Focal up-regulation and activation of the fibronectin receptor component, integrin β1, in the surface ectoderm anchors cells to the fusion site, facilitating junctional remodelling and the establishment of a semi-rosette cellular structure. This cell configuration allows the formation and maturation of novel cell-cell junctions between the opposing surface ectoderm cells, promoting zipper progression and neural tube closure. The integrin-mediated basal anchorage mechanism for fusion and zippering of the mouse neural tube is indispensable since the loss of integrin β1, through either genetic or laser ablation, halted zippering progression, causing failure of neural tube closure. Subsequent research showing that disruption of the integrin regulator TMEM132A causes caudal NTDs in mice further supports the importance of integrin β1 in neural tube closure [54].

Cellular protrusions have long been observed along the apposed edges of the closing neural tube in different fixed model organisms [55–57]. Previously, SEM analysis has revealed the requirement of both membrane-like ruffles and filopodia, suggested to originate from the surface ectoderm, for normal neural tube closure. Additionally, conditional gene knockout experiments illustrated the role of small GTPases, Rac1 and Cdc42, as molecular drivers for cellular protrusions [58]. However, only by pioneering *ex vivo* live imaging of mouse neurulation was it first possible to observe the dynamics of these cellular projections [57,59]. In recent work, live imaging of genetically labelled non-neural ectoderm revealed the involvement of different cellular projections, lamellipodia and filopodia, in promoting neural tube closure [60]. In the cervical spinal region, most cellular projections were identified as lamellipodia while in regions of hindbrain closure the non-neural ectoderm formed primarily filopodia-like cellular projections. High-speed imaging has also revealed the highly dynamic nature of these cellular projections, extending and retracting quickly and in different directions. These results also raise the possibility that filopodial projections may be used to pass positional information onto neighbouring cells allowing cellular protrusions to always be localised directly ahead of the zipping fork. Although these cellular protrusions have been observed for decades, it is still not clear how and why their morphology varies along the rostral-caudal axis, and exactly how they may promote closure of the neural tube. Continuing advances in live imaging technology are likely to provide much more insight into these enigmatic cellular structures.

### Apoptosis

**Figure 3.**
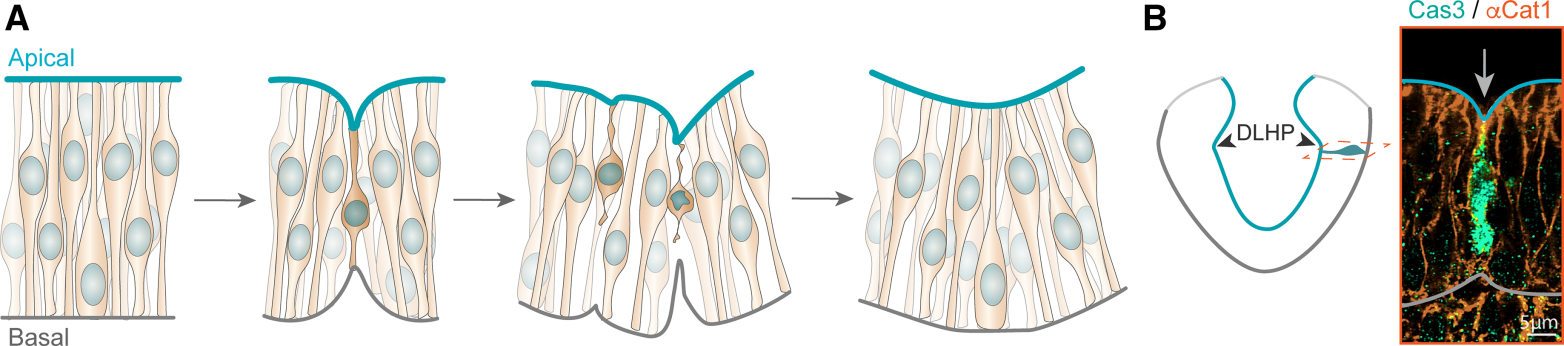
Apoptosis generates mechanical forces to bend the neural plate. (**A**) Schematic showing how apoptosis in the neural plate generates a force that deforms the apical and basal surfaces of the neuroepithelium. This results in the bending of the neural plate, specifically at the dorsolateral hinge points (DLHP). (**B**) Cas3 and alpha-catenin 1 staining reveals the apoptotic cell. The apical and basal surface of the neuroepithelium are outlined. Adapted with permission from [63].

Apoptosis has previously been proposed to be involved in tissue remodelling and neural tube formation, but its exact contribution has remained elusive [61,62]. Recent work using live imaging of chick embryos coupled with cleaved caspase 3 staining suggests that apoptosis may play a mechanical role in the morphogenesis of the dorso-lateral hinge points (DLHP) during neural plate bending (Figure 3) [63].

The incidence of apoptosis increases in future DLHP regions immediately preceding tissue bending, and inhibiting apoptosis prevents bending specifically at the DLHP. During apoptosis, the DLHP cells assemble an apical-basal actomyosin cable which contracts, shortening the cell and deforming the apical surface. Laser ablations demonstrated that the actomyosin cable generates a force that deforms the apical and basal surfaces of the neuroepithelium. This force may also be transmitted to neighbouring cells, changing their shape and imparting a ‘topological memory’ which persists after fragmentation of the apoptotic cell. In this way, the increased incidence of apoptotic cells in the DLHP region may employ a ratchet-like mechanism to progressively bend the neural plate.

### Radial cell intercalation

**Figure 4.**
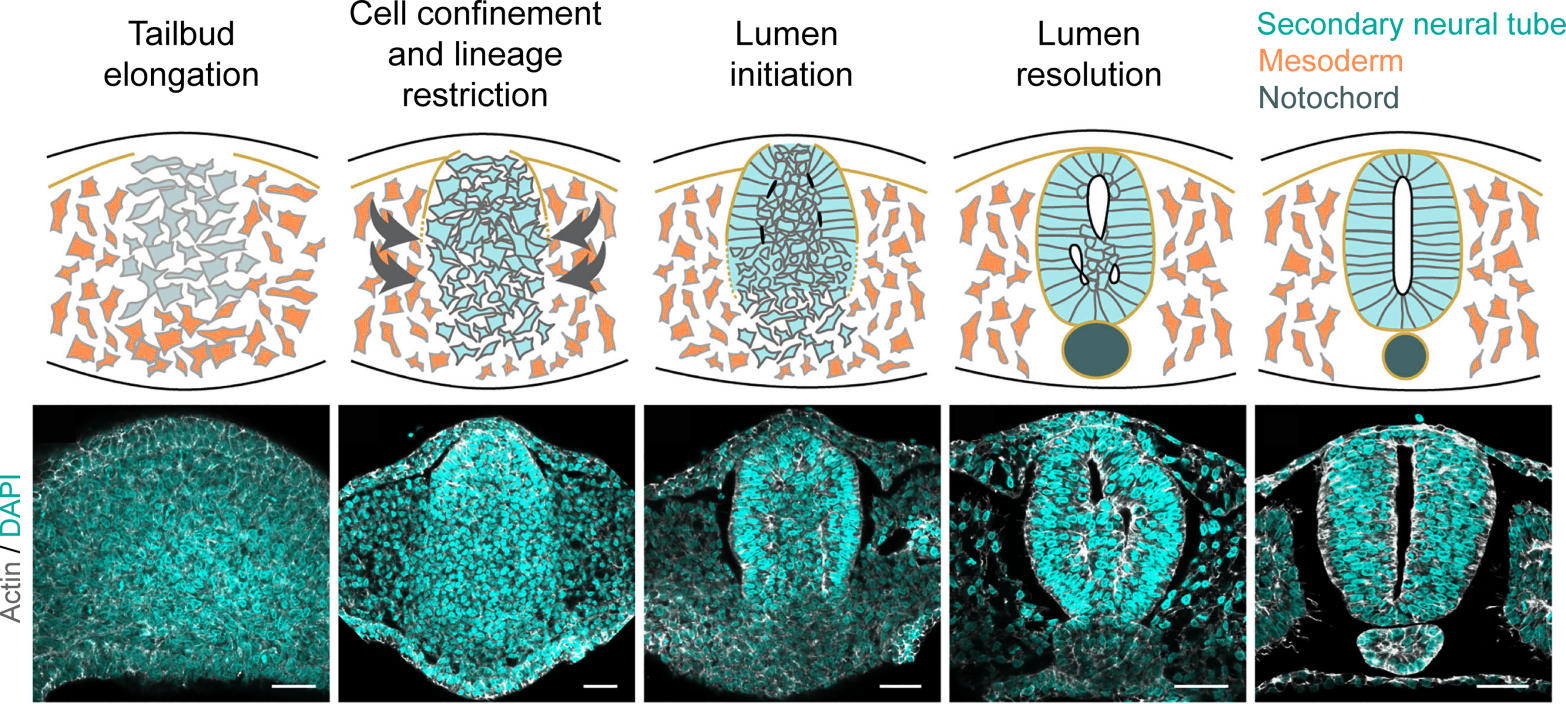
The cellular processes involved in secondary neural tube formation. The tailbud elongates as a result of neuromesodermal progenitor recruitment. The neuromesodermal progenitors become confined and lineage restricted to neural progenitors, upon which lumen formation is initiated to form small cavities. The lumen is resolved to form a single central lumen to complete the process of secondary neurulation. Adapted with permission from [68].

Cell intercalation is the process by which neighbouring cells exchange places to drive morphogenesis during development [64]. With implications for CE and polarised tissue bending, cell intercalation is also thought to be force producing [65,66]. Live imaging in the chicken embryo recently provided the first visualisation of a long-proposed role of cell intercalation in the formation of the secondary neural tube (Figure 4) [67,68]. The final resolution of the central lumen was shown to be mediated by SMAD3-dependent cell intercalation [68]. SMAD3 is a downstream modulator of the TFG-β signalling pathway with known roles in neuronal differentiation and cell fate specification [69]. Together with the cell density-sensing YAP signalling pathway, SMAD3 mediates cell motility to allow the intercalation of central cells into the lateral walls of the neuroepithelium. Inhibition of SMAD3 is associated with a multi-lumen phenotype in the caudal neural tube with YAP overexpression capable of rescuing the phenotype. This suggests that SMAD3/YAP signalling is required for normal lumen resolution during secondary neurulation by mediating the motility of the central cell mass.

## Perspectives

- Understanding the cellular, molecular and mechanical mechanisms of neural tube formation allows for better clinical understanding of NTDs as well as other central nervous system related problems.
- Live imaging in various animal models has revealed common cellular dynamics such as convergent extension, apical constriction and cell intercalation drive morphogenesis of the neural tube. How these dynamic processes vary along the anterior–posterior axis, interact with tissue geometry and generate forces is now beginning to come to light.
- Continuing advancements in live imaging technologies, image analysis and computational modelling will enable a greater understanding of how cellular dynamics are spatially and temporally integrated to shape the developing neural tube.

## Open Access

Open access for this article was enabled by the participation of University of Queensland in an all-inclusive *Read & Publish* agreement with Portland Press and the Biochemical Society under a transformative agreement with CAUL.

## Abbreviations

AC: apical constriction
CE: convergent extension
DLHP: dorso-lateral hinge points
HNP: hindbrain neuropore
NTDs: neural tube defects
PCP: Planar Cell Polarity
PNP: posterior neural pore
